# Development and validation of a physiologically based kinetic model for starting up and operation of the biological gas desulfurization process under haloalkaline conditions

**DOI:** 10.1016/j.wroa.2019.100035

**Published:** 2019-07-02

**Authors:** Karine Kiragosyan, Johannes B.M. Klok, Karel J. Keesman, Pawel Roman, Albert J.H. Janssen

**Affiliations:** a Wetsus, European Centre of Excellence for Sustainable Water Technology, Oostergoweg 9, 8911, MA, Leeuwarden, the Netherlands; b Environmental Technology, Wageningen University, P.O. Box 17, 6700, AA, Wageningen, the Netherlands; c Paqell B.V., Reactorweg 301, 3542, AD, Utrecht, the Netherlands; d Biobased Chemistry & Technology, Wageningen University, P.O. Box 17, 6700, AA, Wageningen, the Netherlands; e Shell, Oostduinlaan 2, 2596, M the Hague, the Netherlands

**Keywords:** Sulfur-oxidizing bacteria, Biological gas desulfurization, Physiologically based kinetics, Flavocytochrome *c*, Sulfide-quinone oxidoreductase

## Abstract

Hydrogen sulfide is a toxic and corrosive gas that must be removed from gaseous hydrocarbon streams prior to combustion. This paper describes a gas biodesulfurization process where sulfur-oxidizing bacteria (SOB) facilitate sulfide conversion to both sulfur and sulfate. In order to optimize the formation of sulfur, it is crucial to understand the relations between the SOB microbial composition, kinetics of biological and abiotic sulfide oxidation and the effects on the biodesulfurization process efficiency. Hence, a physiologically based kinetic model was developed for four different inocula. The resulting model can be used as a tool to evaluate biodesulfurization process performance. The model relies on a ratio of two key enzymes involved in the sulfide oxidation process, i.e., flavocytochrome *c* and sulfide-quinone oxidoreductase (FCC and SQR). The model was calibrated by measuring biological sulfide oxidation rates for different inocula obtained from four full-scale biodesulfurization installations fed with gases from various industries. Experimentally obtained biological sulfide oxidation rates showed dissimilarities between the tested biomasses which could be explained by assuming distinctions in the key-enzyme ratios. Hence, we introduce a new model parameter *α* to whereby *α* describes the ratio between the relative expression levels of FCC and SQR enzymes. Our experiments show that sulfur production is the highest at low *α* values.

## Highlights

• A physiologically based kinetic model was developed for four inocula.
• *α* was introduced to describe inocula potential for sulfur and sulfate formation.
• High *α* values (>0.8) indicate the high potential of inocula for sulfate formation.
• Low *α* values (<0.8) betoken higher sulfur formation.
• *α* is a screening tool for inoculum selection to predict process performance.

## 1 Introduction

During the anaerobic treatment of wastewater, biogas is produced from organic matter (Driessen et al., 2011; Lettinga, 1995). When sulfate is present in the wastewater, this will be converted to sulfide, and a fraction hereof will transfer to the biogas. H_2_S concentrations in the biogas generally range between 100 and 40 000 ppm(v) (Driessen et al., 2011). To be able to use this biogas, strict specifications have to be applied with respect to hydrogen sulfide (H_2_S) levels. In natural gas, the H_2_S concentration has to be below 3 ppm(v). The release of H_2_S to the environment is regulated due to its toxic and corrosive properties (Schnele et al., 2016; World health organization, 2003). Thus, removal of H_2_S is required.

Nowadays, a variety of desulfurization processes are available to remove H_2_S from sour gas streams. Among these technologies, the biological conversion of H_2_S is the most environmentally friendly because no toxic chemicals are required, and the process is operated at ambient conditions, i.e. no high pressures or temperatures. A biotechnological process for the removal of H_2_S was developed in the 1990s, which has been applied in different industrial sectors worldwide (Buisman et al., 1990; Driessen et al., 2011). The process is based on the absorption of H_2_S from sour gas streams in an haloalkaline solvent with a salinity between 0.5 – 2 M Na^+^ and a pH between 8 – 10 (Janssen et al., 2009; Van Den Bosch et al., 2007).

The dissolved bisulfide (HS^-^) is subsequently directed to a bioreactor where haloalkaline sulfur-oxidizing bacteria (SOB) consume reduced sulfur ions and produce elemental sulfur (S_8_) as the end-product (Eq. (1)) (Buisman et al., 1989).

(1) HS^-^ + ½ O_2_ → ⅛ S_8_ + OH^-^

In addition, a small part of the sulfide is oxidized to sulfate according to:

(2) HS^-^ + 2 O_2_ → SO_4_^2-^ + H^+^

Next to biological sulfide oxidation, chemical oxidation can take place:

(3) HS^-^ + O_2_ → ½ S_2_O_3_^2-^ + ½ H_2_O

The formation of sulfur is preferred as hydroxide ions are re-generated, which are required to absorb hydrogen sulfide from the gas stream (Driessen et al., 2011). In addition, the formed sulfur particles can be used as a fertilizer and for sulfuric acid production (A.J.H. Janssen, A. De Keizer, Lettinga, 1994). On the other hand, (thio)sulfate production leads to acidification of the reactor suspension, which requires the addition of sodium hydroxide to maintain the pH for the bacterial optimum conditions and adsorption of sulfide. Hence, in order to optimize the formation of sulfur, the oxygen supply should be carefully controlled (Janssen et al., 1995).

Haloalkaline SOB are naturally occurring microorganisms that can be found in alkaline and highly saline environments, such as soda lakes (Sorokin et al., 2013; Sorokin and Kuenen, 2005). Most known haloalkaline SOB are members of the *Gammaproteobacteria* class, belonging to the genera *Ectothiorodospira*, *Thioalkalivibrio, Thioalkalimicrobium,* and *Thioalkalispira* (Foti et al., 2007). Bacteria from *Ectothiorodospira* genus are phototrophic sulfur purple bacteria, whereas the other three genera are obligate chemolithoautotrophs using various reduced inorganic sulfur compounds as an electron donor (Ghosh and Dam, 2009). SOB can use two groups of enzymes for sulfide oxidation: the periplasmic FAD-containing flavocytochrome *c* (FCC) and the membrane-bound sulfide-quinone oxidoreductase (SQR) donating electrons to the UQ pool (Griesbeck et al., 2000). When (bi)sulfide oxidation is mediated by FCC, (bi)sulfide is oxidized to sulfane (S^0^), using oxidized cytochrome *c* (cyt ^+^) as an electron acceptor (Dahl, 2006; Griesbeck et al., 2000):

(4) HS^-^ + 2 cyt^+^ → S^0^ + 2 cyt + H^+^

Subsequently, the reduced cytochrome *c* (cyt) is oxidized through the reduction of oxygen to water and governed by cytochrome *c* oxidase (Mitchell and Moyle, 1967):

(5) 4 cyt + 4 H^+^ + O_2_ → 4 cyt^+^ + 2H_2_O

However, the role of FCC as the major responsible enzyme for sulfide oxidation has been questioned as many SOB species lack this protein (Kappler, 2007). The SQR pathway is energetically more favorable and less sensitive to inhibition by toxic compounds, for example, methanethiol (Brune, 1989). The SQR mediated sulfide oxidation end-product is a soluble polysulfide (Griesbeck et al., 2002). The SQR route prevails when sulfide oxidation takes place at oxygen-limiting conditions (Klok et al., 2013). In addition, it is postulated that SOB may contain both enzymes and the environmental conditions regulate which enzyme activity prevails. To be able to grow, the haloalkaliphilic chemolithoautotrophic SOB must have specially adapted bioenergetics (Muyzer et al., 2011).

In full-scale gas biodesulfurization installations differences between microbial community compositions were observed (Roman et al., 2016b). Expression of sulfide-oxidizing routes, which define reaction kinetics, and observed bacterial growth rates influence the process efficiency. The aim of this study is to understand the relation between the bacterial community composition, biological sulfide oxidation kinetics and the biodesulfurization process efficiency to optimize sulfur formation.

## 2 Materials and methods

### 2.1 Experimental setup and design

**Fig. 1:**
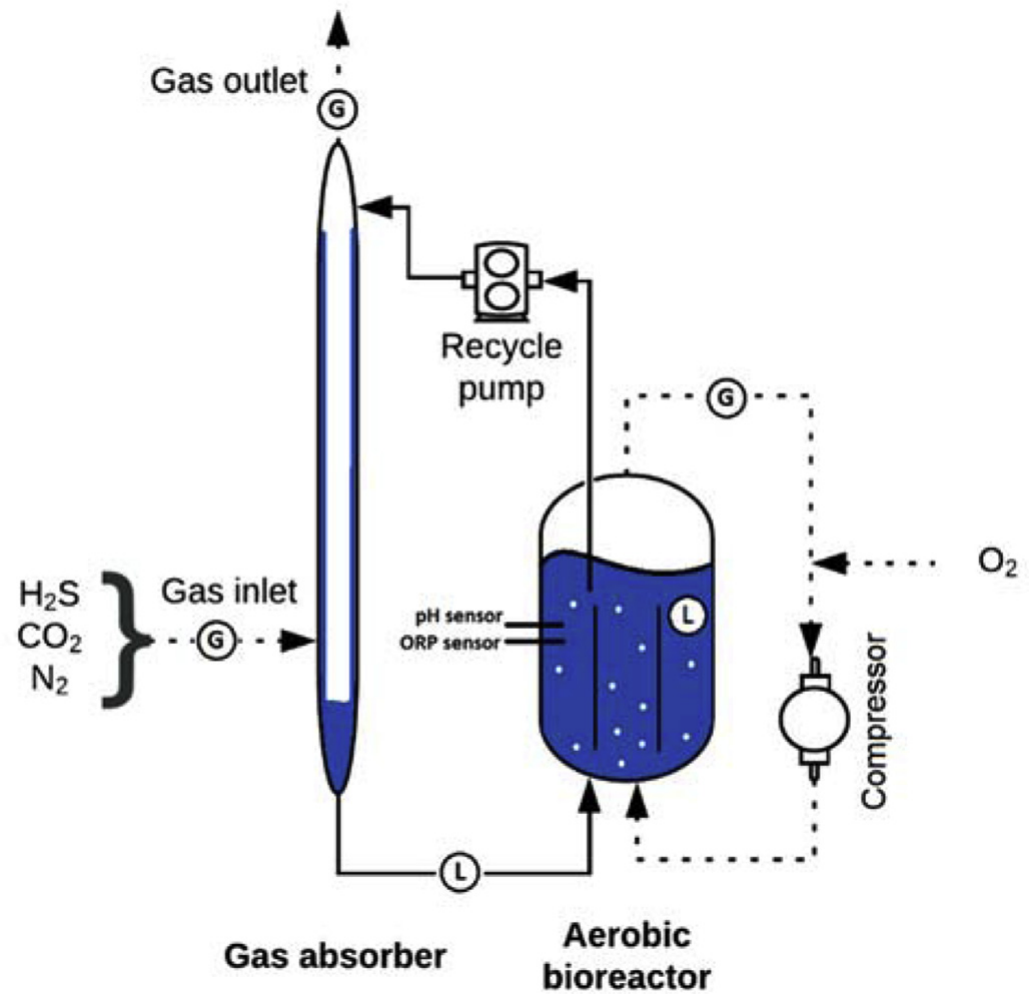
Schematic representation of the experimental setup, operated in the fed-batch mode, used for the experiments (adapted from Roman et al., 2014).

The laboratory setup consisted of a falling film gas absorber integrated with a gas-lift reactor (Fig. 1) (Roman et al., 2016a). Gases were supplied to the gas absorber using mass flow controllers (type EL-FLOW, model F-201DV-AGD-33-K/E, Bronkhorst, the Netherlands). For each gas, a mass flow controller was selected based on the dosing rate, for hydrogen sulfide 0-17 mL min^-1^ was used, for nitrogen 0-350 mL min^-1^, for oxygen 0-30 mL min^-1^ and carbon dioxide 0-40 mL min^-1^. Hydrogen sulfide and nitrogen gas were continuously supplied, whereas the oxygen and carbon dioxide dosing rates were pulse-wise controlled with a multiparameter transmitter (Liquiline CM442-1102/0, Endress+Hauser, Germany) based on the signals from a redox sensor, equipped with an internal Ag/AgCl reference electrode (Orbisint 12D-7PA41; Endress+Hauser, Germany) and a pH sensor (Orbisint 11D-7AA41; Endress+Hauser, Germany). A digital gear pump was used to assure liquid recirculation between the bioreactor and the gas absorber (EW-75211-30, Cole-Palmer, USA) at a constant flow of 0.166 L min^-1^. A gas compressor (N-820 FT.18, KNF Laboport, USA) was used to continuously recycle gas (20 L min^-1^) over the bioreactor. The gas absorber and the bioreactor temperature were regulated at 35 ˚C by a thermostat bath (DC10, Thermo Haake, Germany). The system was sampled in gas and liquid phases. Liquid samples were taken from two sampling points located at the bottom section of the absorber and in the bioreactor (Fig. 1). Gas phase samples were taken from three locations: gas inlet, bioreactor headspace, and absorber outlet.

We conducted four similar experimental runs under stable operating conditions (Table 1) with different inocula (section 2.2). Each experimental run lasted for about six days during which a stable reactor performance was achieved. Sampling was done in technical triplicates at regular time intervals. In our experiments, pH and temperature were kept constant. Oxidation-reduction potential (ORP) set-point value was chosen at -390 mV to suppress sulfate formation (Roman et al., 2016b).

**Table 1:** Overview of the process conditions.

Total liquid volume, L	2.5
pH	8.50 ± 0.05
Salinity, Na^+^ M	1
Temperature, °C	35.0 ± 0.1
H_2_S loading, mM S day-^1^	58.2
ORP set-point, mV	-390

### 2.2 Biomass sources

Biomass samples for inoculation were collected from four different full-scale systems for gas biodesulfurization, which have been in operation for more than ten years. Each biomass was studied separately (one-by-one) under similar experimental conditions. The lab-scale setup was inoculated with cells obtained by centrifugation (15 min at 16 000 x *g*) of a 2.5 L culture collected from full-scale installation. These full-scale systems were selected based on the feed gas composition it treats and on the industry of application.

Table 2 provides a brief overview of the selected installations. Two full-scale systems, which treat sour gas from the anaerobic digestion of the wastewater from the paper pulp industry were sampled. In this paper, the various biomasses will be denoted by the location of the sampling installation.

**Table 2:** A brief description of the origin and averaged operational parameters of the chosen installations.

Location	Industry	Sour gas composition	Sour gas loading, m^3^ h^-1^	ORP set-point, mV	Na^+^, M	K^+^, mM
Eerbeek (NL)^a^	Paper mill	biogas, 0.7% H_2_S	418	-335	0.8	0.7
Zuelpich (DE)^b^	Paper mill	biogas, 0.5% H_2_S	700	-370	0.9	1.5
Amersfoort (NL)	Landfill waste	landfill gas, 0.3% H_2_S	NA	NA	1.3	1.6
Southern Illinois (USA)^c^	Oil and gas	associated gas, 1-5% H_2_S, 50-200 ppm VOSC	800-1100	NA	0.9	3.7

a - (Janssen et al., 2009).

b - (Driessen et al., 2011).

c - (Roman et al*.,* 2016).

### 2.3 Medium composition

The haloalkaline medium for inoculum was buffered with 0.045 M Na_2_CO_3_ and 0.91 M NaHCO_3_. The medium contained 1.0 g K_2_HPO_4_, 0.20 g MgCl_2_ x 6H_2_O and 0.60g urea, each per 1 L of ultrapure water (Millipore, the Netherlands) and trace elements solution as described in Pfennig and Lippert (1966). The pH of the medium was 8.50 ± 0.05 at 35 ˚C. For the respiration test, the medium contained carbonate/bicarbonate buffer only. Trace elements were excluded because they enhance the chemical oxidation of sulfide (Luther et al., 2011).

### 2.4 Respiration test

**Fig. 2:**
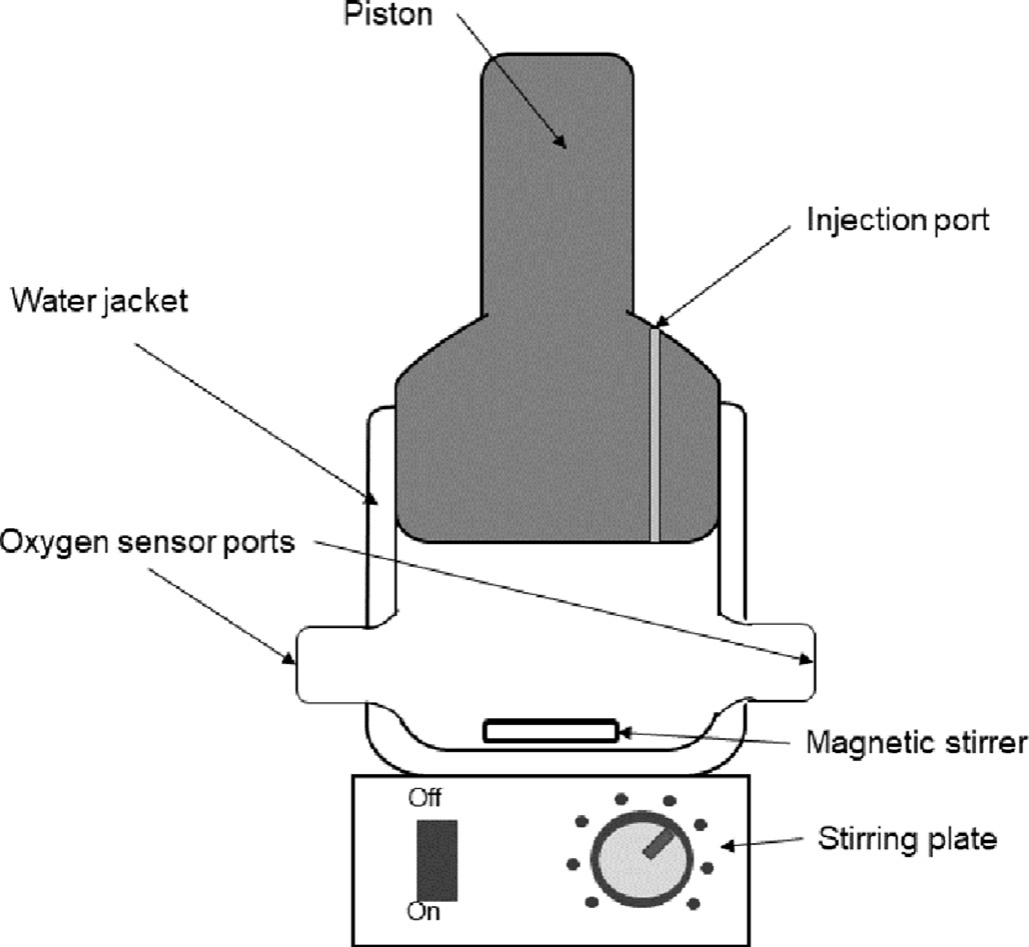
Schematic representation of the thermostated batch reactor used for the respiration tests.

Respiration tests, also known as biological activity monitoring (BOM) tests, were performed to measure biological sulfide oxidation reaction rates in an air saturated medium. A similar setup was used by Roman et al. (2015), consisting of a glass mini-reactor (45 mL), a magnetic stirrer, and a Teflon piston to avoid any oxygen ingress (Fig. 2). Sulfide was added to the reactor, from a freshly prepared stock solution (Na_2_S x 9H_2_O, Sigma Aldrich, the Netherlands), with a glass syringe passing through the piston. The concentration of the prepared stock solution was verified with a sulfide Methylene blue cuvette test (LCK653). If the stock was used for several days, the concentration of the stock was verified every time before use. The sulfide oxidation rate was calculated from measuring the oxygen removal rate with a dissolved-oxygen (DO) sensor (Oxymax COS22D, Endress+ Hauser, Germany). The DO concentration was recorded using a multiparameter transmitter (Liquiline CM442-1102/0, Endress+Hauser, the Netherlands). All experiments were performed at 35 ˚C (DC10, Thermo Haake, Germany) which is in agreement with the conditions in the lab-scale fed-batch setup. As temperature and medium composition were similar to previous studies, a proper comparison of our results can be performed (De Graaff et al., 2012; Klok et al., 2013; Roman et al., 2015; Van Den Bosch et al., 2009).

At the end of each fed-batch bioreactor run the tested biomass was collected to measure specific biomass activities. Biomass was centrifuged and separated from sulfur particles, salts by washing with a 1 M carbonate/bicarbonate buffer. Hereafter biomass was ready to be used for the respiration tests at constant concentration 2 mg N L^-1^. Firstly, biomass was aerated as described elsewhere (Van Den Bosch et al., 2009). Experiments started by injection of sulfide and the initial slope of the recorded oxygen consumption profile was used to calculate the oxygen consumption rate. Biological reaction rates were determined by subtracting the chemical oxidation rates from the measured overall oxygen consumption rates. Chemical rates were measured in the absence of biomass. In addition, we calculated the endogenous oxygen consumption rate based on the respiration measurements without sulfide addition (Van Den Bosch et al., 2009).

### 2.5 Application of a physiologically based kinetic model

The proposed model (Klok et al., 2013) describes both oxidation rates of sulfide through FCC and SQR enzymes, i.e. primary dehydrogenases involved in biological sulfide oxidation, and the effect on end-product formation, i.e. sulfur and sulfate. The calculated maximum sulfide oxidation rate (*μ*) of the involved enzymes was determined by the results from respiration tests.

The electrons released from HS^-^ are transferred to the oxidized form of intermediate acceptors, i.e. either cytochrome *c* or ubiquinones. The reduced co-factors are subsequently oxidized through other enzymes, such as cytochrome *c*-oxidase (CCO) and quinol oxidases (SQRox) (Klok et al., 2013). Irrespective of the type of sulfide dehydrogenase employed, part of the electrons is transferred to oxidized nicotinamide adenine dinucleotide (NAD^+^). The kinetic model contains expressions for the rates of four respiratory enzymes (*μ*_*FCC,*_ *μ*_*SQR,*_ *μ*_*CCO,*_ *μ*_*SQRox*_ in mmol S mg N^-1^ h^-1^) and the associated affinity constants (*K*_*FCC*_*, K*_*SQR*_*, K*_*CCO*_*, K*_*SQRox*_ in mM). In addition, CCO is inhibited by sulfide and therefore an inhibition constant is included (*K*_*i*_ in mM). Lastly, the reduction degree (*F*) of cytochrome *c* and quinone (*Q*) pool is included in the rates equations, which change instantaneously according to sulfide and oxygen levels (i.e. quasi-steady state) (Klok et al., 2013). The rates for the oxidation of dissolved (bi)sulfide and reduction of dissolved oxygen are described by:

(6) $$q_{FCC}=\mu_{FCC}\cdot \left(1-F\right)\cdot \frac{\left[HS^{-}\right]}{K_{FCC}+\left[HS^{-}\right]}$$

(7) $$q_{CCO}=\mu_{CCO}\cdot F\cdot \frac{\left[O_{2}\right]}{K_{CCO}+\left[O_{2}\right]}\cdot \frac{K_{i}}{K_{i}+\left[HS^{-}\right]}$$

(8) $$q_{SQR}=\mu_{SQR}\cdot \left(1-Q\right)\cdot \frac{\left[HS^{-}\right]}{K_{SQR}+\left[HS^{-}\right]}$$

(9) $$q_{SQRox}=\mu_{SQRox}\cdot Q\cdot \frac{\left[O_{2}\right]}{K_{SQRox}+\left[O_{2}\right]}$$

with *q* in mmol S mgN^-1^ h^-1^, $\left[HS^{-}\right]\;$ in mM and $\left[O_{2}\right]$ in mM.

Expression levels for both FCC and SQR were estimated from respiration tests for all tested biomasses described in Table 2. The maximum rates for sulfide oxidation through FCC, i.e. $\mu_{FCC}$ and $\mu_{CCO}$, and SQR, i.e. $\mu_{SQR}\;$and $\mu_{SQRox}$, were estimated using a non-linear least-squares estimation routine. As FCC and SQR expression levels do not describe the reduction of oxygen, it was assumed that increased expression levels of the sulfide-oxidizing enzyme systems would lead to a homologous increase of expression levels of the oxidase enzymes associated with the oxidizing sulfide enzymes, i.e. CCO is associated with FCC and SQRox is associated with SQR. The affinity constants for sulfide and oxygen remained equal to the parameters estimated by Klok et al., (2013) (Table 3). More details concerning the parameter estimation and associated standard deviations can be found in Appendix B.

**Table 3:** Parameters for the physiologically based kinetic model (adapted from Klok et al., 2013).

Affinity constants	
*K*_*FCC*_, mM	0.05
*K*_*SQR*_, mM	1.80
*K*_*CCO*_, μM	2.30
*K*_*SQRox*_, μM	0.23

Inhibition constants	

*K*_*i*_, mM	0.62

The reduction degree of CCO dictates the formation rate of sulfate in the kinetic model. The stronger the oxidation degree of the cytochrome pool (i.e. smaller *F*), the higher the potential for the formation of sulfate (Klok et al., 2013; Visser et al., 1997). We hypothesize that the ratio of expression of oxidation routes of sulfide through either FCC (requiring cytochrome *c* as a cofactor) and SQR (require quinones as a cofactor) is an indicator for the sulfate forming (and thus sulfur forming) potential of SOB under oxygen-limiting conditions. Hence, we postulate that the ratio of $\mu_{FCC}$ and $\mu_{SQR}$ is a predictor of sulfur forming potential. Therefore, we introduce the parameter *α*, defined as $\alpha =\frac{\mu_{FCC}}{\mu_{SQR}}$. Based on the dependencies between sulfate formation, the overall biological activity under oxygen-limiting conditions and the oxidation state of the cytochrome system, we hypothesize that the smaller the value of *α*, the higher the potential for sulfur formation as the end-product.

### 2.6 Analytical techniques

Two types of samples were prepared, i.e. filtrated and precipitated with zinc acetate for anions measurements and non-filtrate for biomass quantification and TOC analysis. All liquid samples were stored at 4 ˚C before being analyzed (about three days).

Biomass quantification was based on the amount of organic nitrogen that was oxidized to nitrate by peroxodisulphate (LCK238 and LCK338, Hach Lange, the Netherlands). The cell pellet was washed twice at 20,238 x *g* for 5 min with the nitrogen-free medium to remove any nitrogen present in the medium.

Sulfate and thiosulfate were measured by ion chromatography (Metrohm Compact IC 761, Switzerland) with an anion column (Metrohm Metrosep A Supp 5, 150/4.0 mm, Switzerland) equipped with a pre-column (Metrohm Metrosep A Supp 4/5 Guard, Switzerland). Immediately after sampling all solids were removed by filtration over a 0.45 μm membrane syringe filter (HPF Millex, Merck, the Netherlands) and mixed with 0.2 M zinc acetate in a 1:1 ratio to prevent any chemical sulfide oxidation. Produced and accumulated sulfur concentration was calculated from the molar sulfur mass balance, which is based on the supplied sulfide load and measured sulfate and thiosulfate concentrations at each sampling time point by following this equation:

(10) d[S^0^] = d(H_2_S supplied/ V_liquid_) – d[SO_4_^2-^] – 2*d[S_2_O_3_^2-^] - S_x_^2-^

Initial sulfur concentration is assumed to be “0”. Concentrations of dissolved sulfide and polysulfides were not taken into account, as their combined contribution to the total concentration of sulfur species is negligible (Kleinjan et al., 2005; Van Den Bosch et al., 2009).

Sulfide and bisulfide were measured as total sulfide (S^2−^_tot_) using the methylene blue method with a cuvette test (LCK653, Hach Lange, USA). Total sulfide quantification was carried out immediately after sampling and samples were diluted in oxygen-free Milli-Q water (sparged with N_2_ stream for 30 min) to exclude chemical sulfide oxidation (Roman et al., 2016c).

In addition to sulfur-containing anions, sodium and potassium concentration were measured with ion chromatography as described earlier (Roman et al., 2015). Using a Metrohm Metrosep C4−150/4.0 mm column with three mM HNO_3_ as the eluent at 0.9 mL min^−1^.

To close the electron balance as described by (Roman et al., 2016b) carbonate and bicarbonate ions concentration were established using the Henderson-Hasselbalch equation (Po and Senozan, 2001). For that, liquid samples were analyzed on total inorganic carbon using high-temperature catalytic oxidation at 680 ˚C with TOC-VCPH/CPN analyzer (Shimadzu, The Netherlands).

The gas phase (H_2_S, N_2_, CO_2,_ and O_2_) was analyzed with a gas chromatograph (Varian CP4900 Micro GC, Agilent, the Netherlands) equipped with two separate column modules, namely a 10-m-long Mol Sieve 5A PLOT (MS5) and a 10-m-long PoraPlot U (PPU).

### 2.7 DNA extraction and 16S rRNA sequencing

Biomass samples were collected for microbial community analysis at the beginning and the end of each experimental run. The samples were washed twice with a buffer solution of pH 8.5 and 0.5 M Na^+^ to prevent the occurrence of an osmotic shock. Afterward, the genomic DNA was extracted from the washed biomass using the DNeasy PowerLyzer PowerSoil Kit (Qiagen) following the manufacturer's instructions. All the above procedures were performed in technical duplicates for each sample, and average values with standard deviations are presented. Library construction and Next-generation sequencing were carried out at the European genome and diagnostics center Eurofins GATC Biotech GmbH (Constance, Germany). The workflow started from 16S rRNA gene amplification in the V3-V5 variable region using 357F (5′- CCTACGGGAGGCAGCAG - 3′) and 926R (5′- CCGTCAATTCMTTTRAGT - 3′) primer set, afterward merging read pairs by overlapping was performed using FLASh (Magoč and Salzberg, 2011) with maximum mismatch density of 0.25. The next step was to cluster sequences based on the similarity with chimera removal with UCHIME (Edgar et al. 2011) using a full length, good quality, and non-chimeric 16S rRNA gene reference database. Cleaned and clustered sequences were BLASTn (Altschul et al. 1990) analyzed using non-redundant 16S rRNA reference sequences with an E-value cutoff of 10^-6^. Only good quality and unique 16S rRNA sequences were taxonomically assigned to the operational taxonomic unit (OTU) to the clusters. The taxonomic classification was based on the NCBI database (www.ncbi.nlm.nih.gov/taxonomy). The EMBL-EBI accession number for presented 16S rRNA sequencing set is PRJEB27163.

## 3 Results

### 3.1 Biodesulfurization process performance

An overview of the results is shown in Table 4. The calculated selectivities for sulfur, sulfate, and thiosulfate are presented as an average value. The term “selectivity” describes the mol fractions of products formed from a reactant or substrate. Detailed information on the obtained experimental data and determination of product selectivities can be found in Appendix C. The lowest selectivity for thiosulfate formation (0.8 ± 0.2 mol%) was obtained for experiments with Landfill biomass and the highest with Paper mill - 1 biomass (17.6 ± 0.3 mol%). Sulfate selectivity was the lowest for Paper mill - 1 system operation (1.1 ± 0.1 mol%), and the highest for Paper mill - 2 and Landfill operation with (7.2 ± 0.4 mol%) and (7.0 ± 0.9 mol%) respectively. The highest sulfur selectivity was achieved with biomass from installations treating Landfill and Oilfield gasses, 92.2 ± 0.9 mol% and 91.0 ± 0.2 mol% respectively.

**Table 4:**
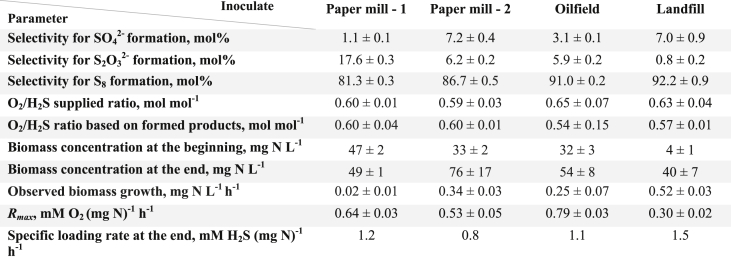
Product selectivity for four different inoculates measured in the lab-scale biodesulfurization set-up for about six days.

The O_2_/H_2_S supply ratio is a critical parameter to control product formation (Van Den Bosch et al., 2009). This parameter can be calculated from the supplied gas flows, as no accumulation of O_2_ nor H_2_S was observed, indicating that all supplied compounds are indeed were consumed. The O_2_/H_2_S supply ratio is compared with the value obtained from the formed products based on the reaction's stoichiometry. The electron balance was validated by comparing the O_2_/H_2_S ratios versus the formed products and that no significant differences were found (Table 4). Hence, we conclude that the mass balance for sulfur compounds is closed albeit that at very low concentrations compounds could be formed that were not analyzed by us. We studied the rates of underlying biological and chemical reactions by performing respiration tests to better understand the formation of various end-products. Therefore, biological kinetic rates were measured using respiration tests. Our results show that the highest maximum biological oxidation rate (*R*_*max*_) was achieved with Oilfield biomass 0.79 ± 0.03 mM O_2_ (mg N)^-1^ h^-1^, and the lowest *R*_*max*_ value was achieved with Landfill biomass 0.30 ± 0.02 mM O_2_ (mg N)^-1^ h^-1^. Nevertheless, both biomasses showed about 90 mol% of sulfur formation in the lab-scale experiments. In addition, the specific substrate loading rate of bacteria is in the same order of magnitude. Hence, the achieved end-product selectivities cannot be solely explained by *R*_*max*_. Next to maximum rates, the observed reaction kinetics are controlled by substrate affinities (Marangoni, 2003). In respiration tests, oxygen levels are typically elevated (100% of DO), i.e. $\left[O_{2}\right]\gg K_{CCO}$, whereas in gas biodesulfurization process DO levels are below the detection limit, i.e. $\left[O_{2}\right]\ll K_{CCO}$. Hence, we have applied a physiologically based kinetic model to describe sulfide oxidation under oxygen-limiting conditions. Moreover, to correlate biological kinetics obtained from respiration tests to values measured in the biodesulfurization process, parameter α was introduced. This parameter is defined as $\alpha =\frac{\mu_{FCC}}{\mu_{SQR}}$, and indicates the relative expression levels of sulfate formation routes. High relative expression levels of the *μFCC* and CCO resulted in higher production of sulfate, which in turn is responsible for cytochrome c pool re-generation. Whereas high levels of the *μSQR* yield in the high formation of sulfur (Klok et al., 2013).

**Fig. 3:**
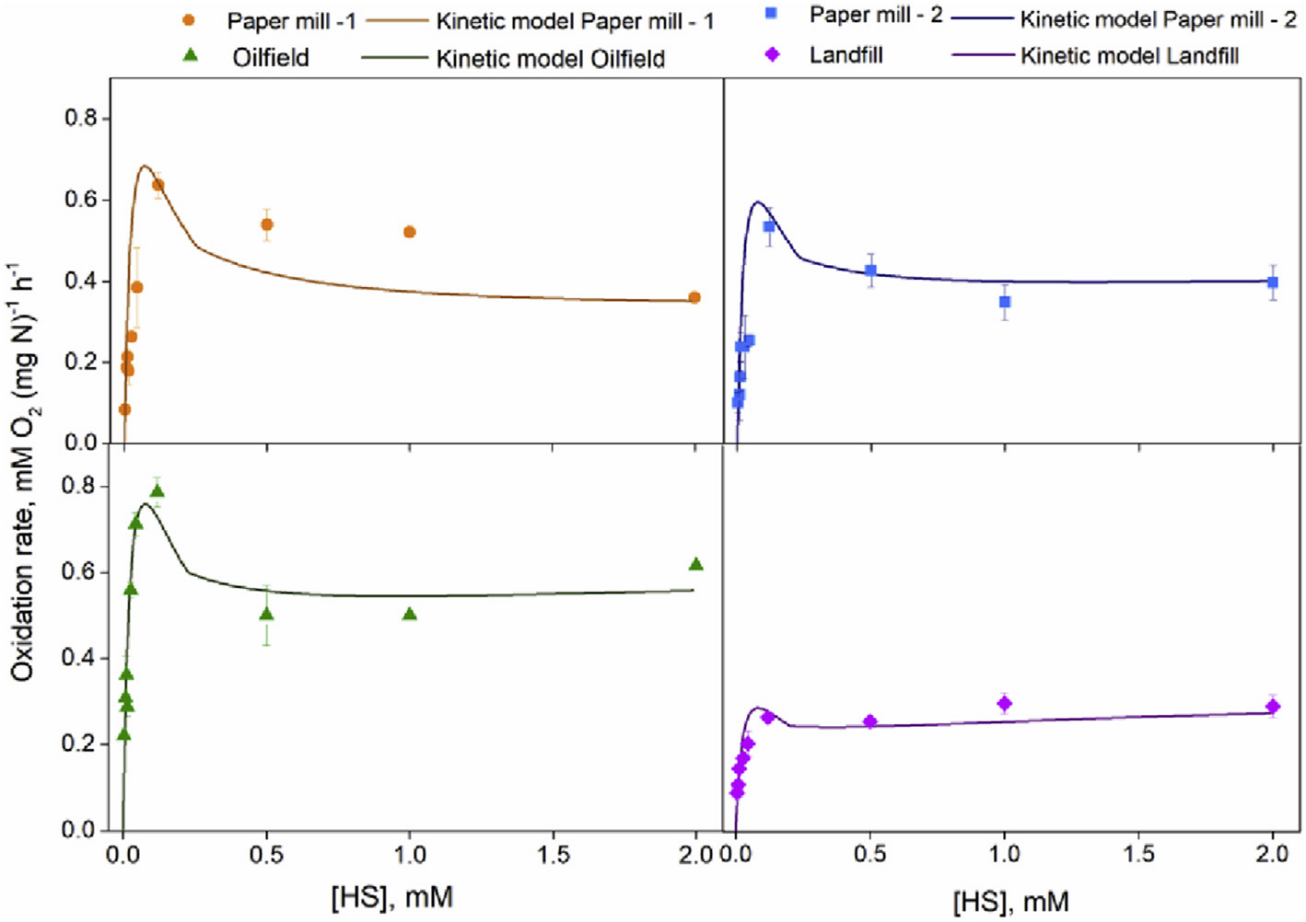
Biological oxidation rates at different concentrations of HS^-^ in oxygen saturated buffer at pH 8.5, 1 M Na^+^ and 35 ˚C. Measured data points are average values of the experimentally measured duplicates. The solid lines indicate the estimated physiologically-based kinetic model.

The parameters in the physiologically-based model proposed by Klok et al. (2013) were recalibrated for four inocula originated from full-scale installations based on the obtained respiration data (Table 5, Fig. 3). Results show that Paper mill - 1 biomass has a high potential for sulfate formation (*α* at 1.23 ± 0.17). Hence, using Paper mill - 1 biomass under oxygen-limiting conditions (ORP -390 mV) results in low biomass activity and consequently in high chemical oxidation rates (17.6 ± 0.3 mol%). The other three biomasses showed significantly lower *α* values, indicating a higher potential for sulfur formation under oxygen-limiting conditions. In addition, the calibrated model was used to predict sulfur selectivities for four tested biomasses at various oxygen concentrations (Appendix D).

**Table 5:**
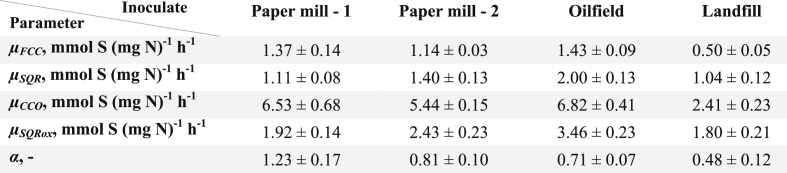
Parameters calculated by the physiological kinetic model.

*μ* is the maximum sulfide oxidation rate for the enzymes FCC, SQR, CCO, and SQR_ox_ respectively *α* the ratio between rates of *μ*_*FCC*_ and *μ*_*SQR*_.

### 3.2 Bacterial community analyses

**Fig. 4:**
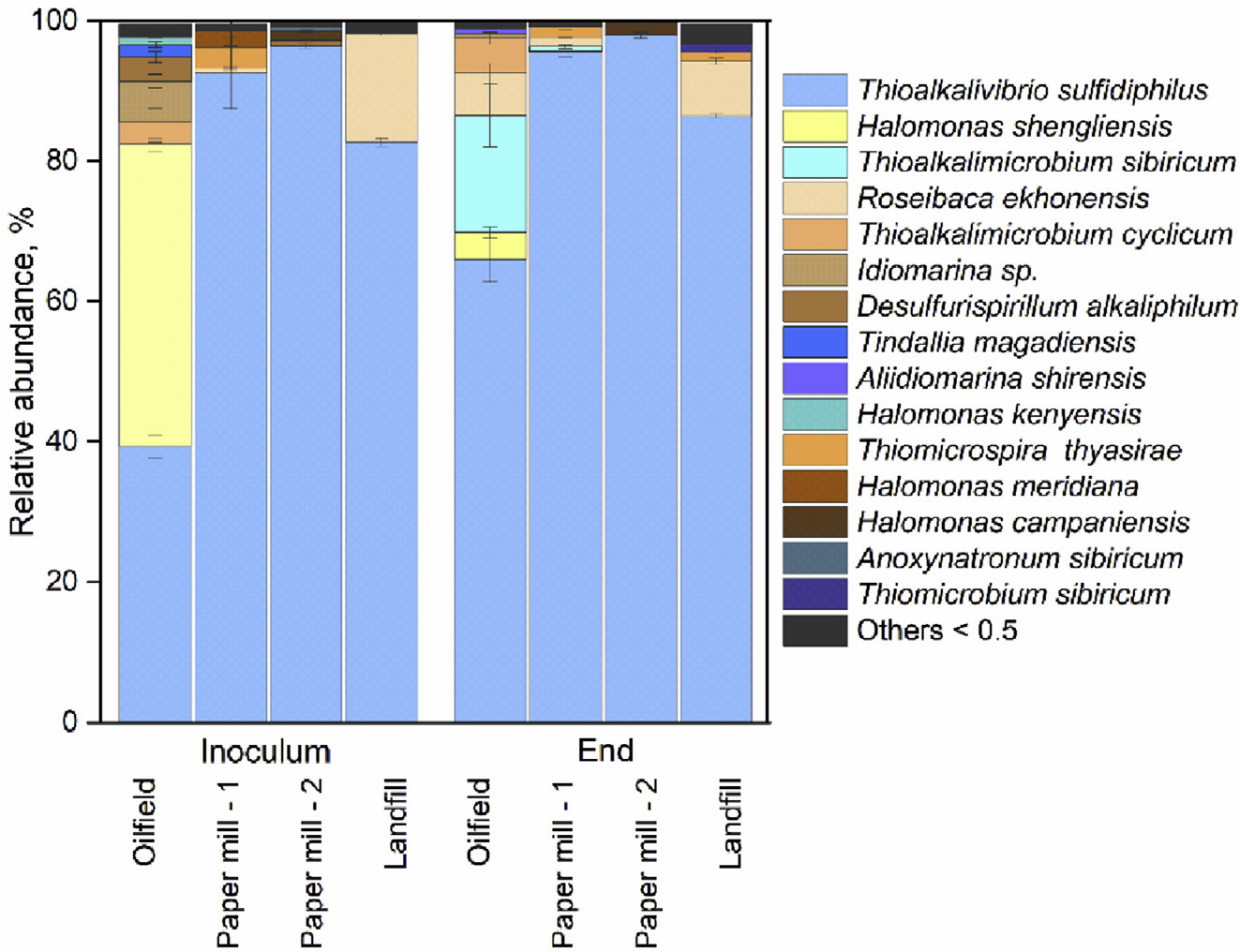
Microbial composition profile of OUTs showing the relative abundance of the species in the inoculum and at the end of each experiment. Oilfield, Paper mill - 1, Paper mill - 2, and Landfill denote biomass origin. Only bacteria with a relative abundance higher than 0.5% are listed (remaining species are clustered into “Others”).

Total DNA was extracted and analyzed using next-generation sequencing of the 16S rRNA gene, at the beginning and at the end of the experimental runs to enable monitoring of the microbial community change. The most dominant species of the microbial communities in the Paper mill - 1, Paper mill - 2 and Landfill inocula were *Thiolalkalivibrio sulfidiphilus* with an estimated abundance of 92.6%, 96.5%, and 82.7%, respectively (Fig. 4). In contrast, in the Oilfield inoculum, a heterotrophic gammaproteobacterium *Halomonas shengliensis* was the most abundant species with 43.1% in comparison to 39.3% of *Tv. sulfidiphilus* (Fig. 4). *Halomonas* species become abundant when organic hydrocarbons are present in the feed streams (Peyton et al., 2004). Oilfield biomass is fed by a gas stream originating from crude oil extraction, which can explain the presence of *Halomonas* species. In Landfill biomass, the second dominant species was an anoxygenic purple nonsulfur producing alphaproteobacterium *Roseibaca ekhonensis* with 15.5% abundancy, whereas its population decreased by a factor of two at the end of the process operation. The least abundant in Paper mill - 1 biomass inoculum were lithoautotrophic SOB *Thiomicrospira thyasirae* and heterotrophic *Halomonas meridiana* with only 3% and 2.3% respectively. In Paper mill - 2 prominent biomass species were *Halomonas campaniensis* 1.3%, and two haloalkaliphilic anaerobes (0.7% each) – sulfur-reducing *Desulfurispirillum alkaliphilum* and fermentative clostridium *Anoxynatronum sibiricum. Desulfurispirillum* has been described previously as a dominant sulfur-reducing bacterium in the Eerbeek plant (Paper mill - 1) (Sorokin et al., 2007), while a close relative of *Anoxynatronum sibiricum* has been enriched and isolated in pure culture from Eerbeek plant in 2009 using thiosulfate as electron acceptor (Sorokin, unpublished results). This indicates that a full sulfur cycle might be functional in micro-aerophilic biodesulfurization bioreactors maintaining highly negative redox potential.

Minor changes in the microbial composition were noticed in the samples collected at the end of the experiments with Paper mill - 1, Paper mill - 2 and Landfill biomass. In Landfill biomass, *R. ekhonensis* abundance decreased to 7.8%, but the other two species *Thiomicrospira thyasirae* and *Thioalkalimicrobium sibiricum* became detectable with 1.3% and 1.2%. Microbial compositions in Paper mill - 1 and Paper mill - 2 biomass at the end of the experiments were similar to the inoculum. In contrast, the Oilfield biomass underwent a profound shift in the microbial community during the performed experiments: the population of *Halomonas shengliensis* decreased from 43.1 to 3.8% and was overtaken by lithoautotrophic *Thiolalkalivibrio sulfidiphilus* (66%). Also, two other haloalkaliphilic SOB species proliferated - *Thioalkalimicrobium sibiricum* and *Roseibaca ekhonensis* with 16.6% and 6.0%, respectively. Changes in the microbial composition of the Oilfield biomass are probably caused by a change in the feed gas composition that was lacking an organic carbon source.

## 4 Discussion

From our experiments, it can be seen that the sulfur selectivity was above 90 mol% for biomasses that originates from Oilfield and Landfill full-scale installations whilst the consortia that come from Paper mill - 1 shows lower sulfur selectivity and a significantly higher thiosulfate formation. In gas biodesulfurization systems thiosulfate is usually formed chemically when the enzymatic oxidation capacity is limited. Thus, it can be used as an indication of limited biological oxidative capacity (Jørgensen and Bak, 1991). Chemically formed thiosulfate can be further oxidized to sulfate by SOB (Sorokin et al., 2008). However, in our lab-scale gas biodesulfurization set-up thiosulfate only accumulated in the process liquid when the abiotic formation rates of thiosulfate were higher than the biological oxidation rates.

To understand the observed differences in formed end-products by different biomasses, we investigated the underlying biological reaction mechanism and kinetics, such as maximum biological respiration rates. Our results of the kinetic experiments are in good agreement with reported literature. Our measurements of *R*_*max*_ (0.64 mM O_2_ (mg N)^-1^ h^-1^) corresponds to data reported by Van Den Bosch et al. (2009), Klok et al. (2012) and Roman et al. (2016c), who tested Paper mill - 1 biomass in their studies and observed *R*_*max*_ in the order of 0.3 – 0.6 mM O_2_ (mg N)^-1^ h^-1^. Differences between these reported values can be explained by fluctuations in the operating conditions over time. For example, at Paper mill - 1 we learned that the solutions’ pH buffer capacity, sulfide concentration in the gas feed, and ORP set-point fluctuated significantly in the period before the inoculum was collected (personal communication with the plant manager). It is known that variations in ORP set-point value in time will vary the oxygen supply rates and thus the O_2_/H_2_S ratio. This, in turn, will affect the selectivities for the various end-products (Van Den Bosch et al., 2007). For example, in the work of Roman et al. (2015) Paper mill - 1 inoculum was also used. In their studies, thiosulfate selectivity was reported two times lower than found in this study. A possible explanation is the observed operational fluctuations (since 2016) at the Paper mill - 1 full-scale installation that affected the potential of the biomass for sulfate and sulfur formation at different ORP set-points.

**Fig. 5:**
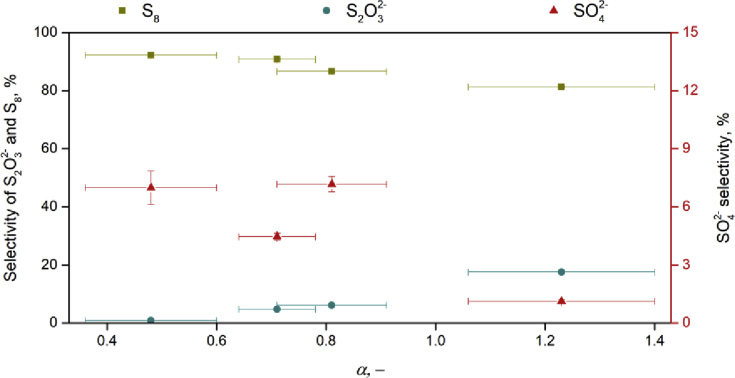
Relationships between sulfur products selectivity and enzymatic ratio *α*.

It can be expected that changes in the biological activity are explained by the differences in microbial physiology. In this study, parameter *α* is introduced to link physiology of biological sulfide oxidation and formation of end-products in the biodesulfurization process. In Fig. 5, the relation between the formed products and *α* is presented for systems operated at oxygen-limiting conditions (ORP = -390 mV). It can be seen that the highest selectivity for sulfur formation (92.2 mol%) was found for the lowest *α* values, i.e. 0.35 – 0.7, whilst the highest selectivity for sulfate formation (7.2 mol%) was found for the highest *α* value (above 0.8). The highest *α* was found for Paper mill - 1 biomass (1.23 ± 0.17), which correlates to a high potential for sulfate formation. However, under oxygen-limiting conditions Paper mill - 1 biomass has low biomass activity. Thus, the formation of thiosulfate is high (17.6 mol%), and sulfate is almost not formed due to oxygen limitation. From these, it follows that *α* can be an effective parameter to screen biomasses, which are able to generate elemental sulfur under oxygen-limiting conditions.

Higher biomass growth rates were found at increasing selectivities for sulfate formation because more energy is liberated from sulfide oxidation compared to the sulfur formation (Buisman et al., 1991; Janssen et al., 1998; Klok et al., 2013). However, growth rates are also dependent on the microbial community composition, as different species have different growth rates and oxidation capacities. For example, the highest measured biomass growth (0.52 ± 0.03 mg N L^-1^ h^-1^) was observed for Landfill biomass, but the highest measured selectivity for sulfate production (7.2 ± 0.4%) was observed for Paper mill - 2 biomass (Table 4). As well as, growth rates of Oilfield and Paper mill - 2 are similar, but selectivity for sulfate is two-fold different. This deviation is possibly caused by the abundance of *Thioalkalimicrobium sibiricum* in Oilfield biomass. *Thioalkalimicrobium* species are known for their high capacity for sulfide oxidation and fast but inefficiently opportunistic growth during short periods of substrate excess (Sorokin and Kuenen, 2005). In contrast, highly abundant *Thioalkalivibrio* species in Paper mill - 1 are slow growing with high growth yield and survive longer during substrate limitation (Sorokin et al., 2007). Hence, in the absence of sulfate formation, the relatively low energy yield from sulfide oxidation was used for cell maintenance rather than Paper mill - 1 biomass growth.

To deepen our understanding of the process performance, a relation between microbial composition and process conditions need to be established. Microbial community composition was determined with 16S rRNA amplicon sequencing and showed that *Thioalkalivibrio sulfidiphilus* was the dominant SOB species in samples from Paper mill - 1, Paper mill - 2, and Landfill. Also, Sorokin et al. found that *Thioalkalivibrio sulfidiphilus* was dominant in Paper mill - 1 (2012). The gas composition fed to the Paper mill - 1 and Paper mill - 2 full-scale plants are almost the same, but operating conditions differ (Table 2). Microbial composition of the Landfill biomass was different from that to Paper mill - 1 and Paper mill - 2. It is known that feed gas composition at landfill installations contains hydrocarbons impurities (Bove and Lunghi, 2005). Hence, it possibly triggered a shift in the microbial composition of the Landfill biomass. A second dominant species in the inoculum is *Roseibaca ekhonensis* described as marine aerobic, heterotrophic and alkalitolerant alphaproteobacterium (Labrenz et al., 2009), which also might have taken advantage of the presence of organic compounds in the Landfill plant. As supplied gas composition in the lab-scale setup differs from the full-scale installation, we observe a microbial composition shift with the reduction of heterotrophs in favor of chemolithoautotrophic SOB.

In comparison to the three tested biomasses, the Oilfield original community changed the most (Fig. 4). Inoculum from the full-scale Oilfield plant contained about 43.1% of *Halomonas shengliensis* – alkalitolerant heterotroph capable of utilizing hydrocarbons that are present in the feed gas (Wang et al., 2007). Its relative abundance drastically decreased as feed gas composition at lab-scale biodesulfurization system contained sulfide only. Second dominant species was *Tv. sulfidiphilus* with an abundance of 39.3%. It is known that *Tv. sulfidiphilus* is the most dominant SOB in gas biodesulfurization bioreactors when the only sulfide is supplied (Roman et al., 2016a; Sorokin et al., 2008). Thus, at the end of the process operation abundance of *Tv. sulfidiphilus* increased (65%). In addition, a fast-growing haloalkaliphilic SOB *Thioalkalimicrobium sibiricum* also proliferated as it grows in the presence of thiosulfate and sulfide (Sorokin et al., 2001).

## 5 Conclusions

In this work, we show that *α* can be used as a screening parameter that is applied for the biomass selection in order to predict process performance. Thus, to achieve desired products formation Factor *α* represents the ratio between the rates of two enzymatic routes for sulfide oxidation. We found that this parameter is a good indicator for the assessment of the end-product formation under oxygen-limiting conditions. In practice, this means that the biomass composition is linked to the process performance and sudden changes in process conditions (e.g., mixing) will not instantaneously change the S_8_ forming potential of the biomass. In addition, *α* will be more determined by the process conditions rather than the bacterial community composition, as process conditions will eventually structure the community composition.Moreover, using process parameters, such as oxygen and sulfide concentration, together with biomass concentration and its activity, its possible to predict the relative formation of biological end-products: sulfate and sulfur. Despite showed variations in four tested biomasses at the inoculum stage, it is expected that under the similar experimental conditions all microbial communities will converge to a similar end composition. We further calibrated an existing kinetic model based on the measured sulfide oxidation rates in batch experiments. The kinetic model relies on a ratio of two key enzymes involved in sulfide oxidation, i.e. flavocytochrome *c* and sulfide-quinone oxidoreductase (FCC and SQR). The updated kinetic model can be used as a tool to evaluate process performance, estimate relative formation of biological end-products, and as an indicator, to select inocula for full-scale installations.
