# Supplementary material for: Development and validation of a physiologically based kinetic model for starting up and operation of the biological gas desulfurization process under haloalkaline conditions
Source: Water Res X. 2019 Jul 2;4:100035. doi: 10.1016/j.wroa.2019.100035 (PMC6614595; doi:10.1016/j.wroa.2019.100035)
Supplement: Multimedia component 1 [file mmc1.docx]

**Appendix A.**

**Table A.1**

Nomenclature

| Symbol | Parameter |
| --- | --- |
| HS^-^ | Bisulfide anions |
| H_2_S | Sulfide |
| SO_4_^2-^ | Sulfate anions |
| S_2_O_3_^2-^ | Thiosulfate anions |
| S_8_ | Biological sulfur |
| O_2_ | Oxygen |
| *R_max_* | Maximum reaction rate |
| SOB | Sulfur-oxidizing bacteria |
| FCC | Flavocytochrome *c* |
| SQR | Sulfide-quinone reductase |
| *µ_FCC_* | Maximum enzyme rate of flavocytochrome *c* |
| *µ_SQR_* | Maximum enzyme rate of sulfide-quinone reductase |
| *µ_CCO_* | Maximum enzyme rate of cytochrome *c* oxidase |
| *µ_SQRox_* | Maximum enzyme rate of sulfide: quinone reductive oxidase |
| *α* | Ratio between maximum enzyme rates of flavocytochrome *c* and sulfide-quinone reductase |
| *K_FCC_* | Affinity constant for flavocytochrome *c* |
| *K_SQR_* | Affinity constant for sulfide-quinone reductase |
| *K_CCO_* | Affinity constant for cytochrome *c* oxidase |
| *K_SQRox_* | Affinity constant for sulfide-quinone reductive oxidase |
| *K_i_* | Affinity constant for inhibition |

**Appendix B.**

Non-linear least squares estimation of the kinetic model describing biological sulfide oxidation.

The unknown parameters in the kinetic model for sulfide oxidation under halo-alkaline conditions, represented by the parameter vector$\theta$, are estimated using the experimental data of the respiration test also known as biological oxygen monitoring tests. Via a least square routine, the estimated single-output gives:

Eq. (B.1) $\hat{\theta}_{N}={\arg min}_{\theta\epsilon D}\sum_{k=1}^{N} {\varepsilon\left( {[{HS}^{-}]}_{k} | \theta\right)}^{2}$

Where $\varepsilon\left( \cdot| \theta\right)=y\left( k \right)-\hat{y}\left( \cdot| \theta\right)$ is the output error at time index $k$ with sulfide concentration$[{HS}^{-}]$, $y\left( k \right)$ the measured sulfate / thiosulfate concentrations at $k$, $\hat{y}\left( \cdot| \theta\right)$ the predicted model output at $k$ given estimate of $\theta$ ($\hat{\theta}$), $D$ is the prior parameter domain. The error variance${\sigma_{\varepsilon}}^{2}$, a measure for the model fit, is given by

Eq. (B.2) ${\sigma_{\varepsilon}}^{2}=\frac{1}{N-p}\sum_{k=1}^{N} {\varepsilon\left( {[{HS}^{-}]}_{k} | \theta\right)}^{2}$

With $p$ the number of parameters. The vector with standard deviations for each parameter is found, after taking the square root of the diagonal of the covariance matrix of the estimates ($COV$), which is defined by

Eq. (B.3) $COV\hat{\theta}_{N}={\sigma_{\varepsilon}}^{2}{(X^{T}X)}^{-1}$

Where $X$ is the (*N* x *p*) sensitivity matrix with elements $\frac{\partial\varepsilon\left( {[{HS}^{-}]}_{k} | \theta\right)}{\partial\theta_{j}}$ with *k* = 1, …, *N* and *j* = 1, …, *p*. While the standard deviation for the µ_FCC_ and µ_SQR_ follow from the estimation routine, the standard deviation for the parameter α=$\frac{\mu_{FCC}}{\mu_{SQR}}$ is given by

Eq. (B.4) ${\sigma_{\alpha}}^{2}=\left( \frac{\partial\alpha}{{\partial\mu}_{SQR}} \right)^{2}\cdot{{\sigma\mu}_{SQR}}^{2}+\left( \frac{\partial_{\alpha}}{{\partial\mu}_{FCC}} \right)^{2}\cdot{{\sigma\mu}_{FCC}}^{2}$

Which results in

Eq. (B.5) ${\sigma_{\alpha}}^{2}=\left( \frac{\mu_{FCC}}{\mu_{SQR}} \right)^{2}\cdot{{\sigma\mu}_{SQR}}^{2}+\left( \frac{1}{\mu_{SQR}} \right)^{2}\cdot{{\sigma\mu}_{FCC}}^{2}$

**Appendix C.**

Non-linear least squares estimation of formation rates.

The formation rate of both SO_4_^2-^ and S_2_O_3_^2-^ in the fed-batch experiments were estimated via a linear regression model given by:

Eq. (C.1) $\hat{y (\theta)}=\theta\left( 1 \right)\cdot x1+\theta\left( 2 \right)\cdot x2$

Where $\hat{y (\theta)}$is the vector containing the predicted model outputs of either sulfate or thiosulfate, $\theta\left( 1 \right)$ the formation rate of sulfate / thiosulfate, $\theta\left( 2 \right)$ the estimated initial concentration of sulfate / thiosulfate, $x1$ a vector of length N containing sampling times and $x2$ a vector of ones with length *N*.

The unknown parameters vector [*θ* (1) *θ* (2)]^T^ was estimated using a non-linear estimation routine, which for the single-output case gives

Eq. (C.2) $\hat{\theta}_{N}={\arg min}_{\theta\epsilon D}\sum_{k=1}^{N} {\varepsilon\left( k | \theta\right)}^{2}$

Where $\varepsilon\left( \cdot| \theta\right)=y\left( k \right)-\hat{y}\left( \cdot| \theta\right)$ is the output error at time index $k$ with operation time $x1$, $y\left( k \right)$ the measured sulfate / thiosulfate concentrations at $k$, $\hat{y}\left( \cdot| \theta\right)$ the predicted model output at $k$ given an estimate of $\theta$ ($\hat{\theta}$), $D$ is the prior parameter domain. The error variance ${\sigma_{\varepsilon}}^{2}$, a measure for the model fit, is given by

Eq. (C.3) ${\sigma_{\varepsilon}}^{2}=\frac{1}{N-p}\sum_{k=1}^{N} {\varepsilon\left( {x1}_{k} | \theta\right)}^{2}$

With $p$ the number of parameters. The vector with standard deviations for each parameter are found, after taking the square root of the diagonal of the covariance matrix of the estimates ($COV$), which is defined by

Eq. (C.4) $COV\hat{\theta}_{N}={\sigma_{\varepsilon}}^{2}{(X^{T}X)}^{-1}$

Where $X$ is the (*N* x *p*) sensitivity matrix with elements $\frac{\partial\varepsilon\left( {x1}_{k} | \theta\right)}{\partial\theta_{j}}$ and with *k* = 1, …, *N* and *j* = 1, …, *p*, in case of A.1 *X* = [*x*1 *x*2]. While the standard deviation for both sulfate ($\sigma_{sulfate}$) and thiosulfate ($\sigma_{thiosulfate}$) follows from the estimation routine, the selectivity for sulfur formation follows from the mass balance according to:

dS^0^ = (dH_2_S_supplied_/V_liq_) – dSO_4_^2-^ – dS_2_O_3_^2-^

As a result, the standard deviation for sulfur formation ($\sigma_{sulfur})$ can be determined from the variance:

Eq. (C.5) ${\sigma_{sulfur}}^{2}={\sigma_{sulfate}}^{2}+{\sigma_{thiosulfate}}^{2}$

The experiment using the Oilfield seed sludge as well as Paper mill - 1, consisted out of two runs with a small interruption due to the system maintenance and medium refreshment. The selectivity for sulfate and thiosulfate was estimated as an average of both experiments. Hence, the standard deviation for both sulfate as thiosulfate can be calculated from the variance

Eq. (C.6) $\sigma^{2}=\frac{{\sigma_{run1}}^{2}+{\sigma_{run2}}^{2}}{2}$

The standard deviation of sulfur follows subsequently from Eq. (C.5). In the following subsections, for each experiment a regression model is identified. The corresponding estimates with standard deviations can be found in Table 4.

**Appendix D.**

To be able to predict the formation of biological products of sulfide oxidation (sulfate and sulfur) it is essential to know *ɑ* value that was calculated based on the performed respiration tests. In addition, it is required to know the concentration of biomass, sulfide loading, and ORP set-point, as oxygen concentration will be determined by ORP value. More details can be found in (Klok et al., 2013).

In this supplementary material, we present three figures. On these figures, we predict sulfate and sulfur selectivity at different oxygen concentrations for four analyzed biomasses. For the prediction we consider *ɑ* values calculated for each biomass, biomass concentration (see Table 4), sulfide loading rate (58.2 mM S day^-1^) and ORP set-point (-390 mV).





**Fig. D.1** Predicted sulfate and sulfur selectivities at high oxygen concentration (200 nM).





**Fig. D.2** Predicted sulfate and sulfur selectivities at elevated oxygen concentration (100 nM).

**

**

**Fig. D.3** Predicted sulfate and sulfur selectivities at limiting oxygen concentration (10 nM).

**Appendix E.**


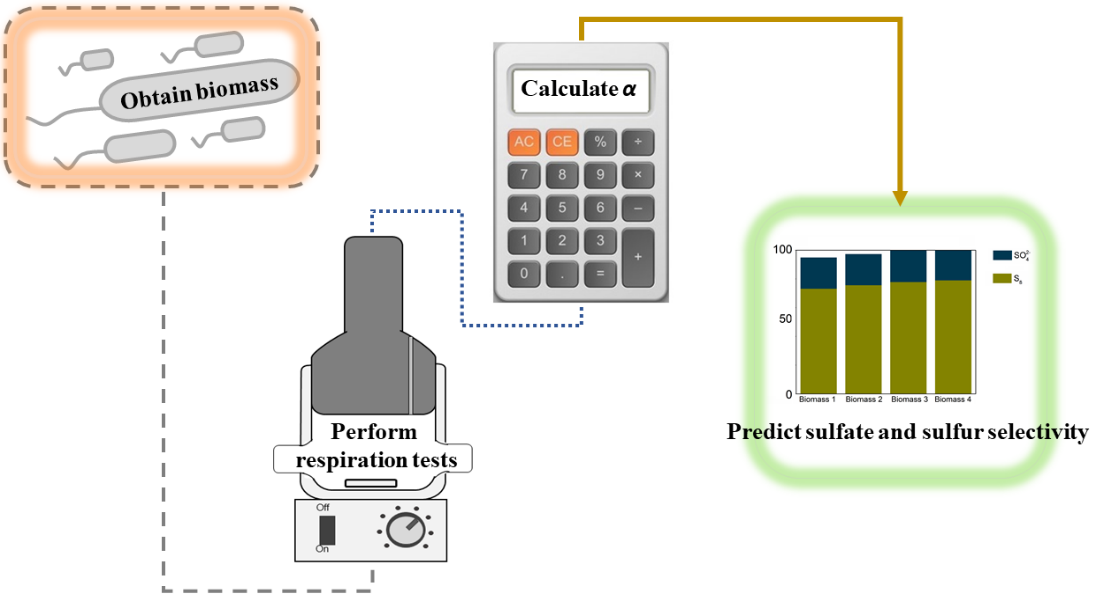


**Fig. E.1** The workflow to screen biomass to achieve desired product formation.
